# Sigma 1 receptor regulates ERK activation and promotes survival of optic nerve head astrocytes

**DOI:** 10.1371/journal.pone.0184421

**Published:** 2017-09-12

**Authors:** Jing Zhao, Barbara A. Mysona, Jing Wang, Graydon B. Gonsalvez, Sylvia B. Smith, Kathryn E. Bollinger

**Affiliations:** 1 James and Jean Culver Vision Discovery Institute, Augusta, Georgia, United States of America; 2 Department of Ophthalmology, Medical College of Georgia, Augusta University, Augusta, Georgia, United States of America; 3 Department of Cellular Biology and Anatomy, Medical College of Georgia, Augusta University, Augusta, Georgia, United States of America; Universidade Federal do Rio de Janeiro, BRAZIL

## Abstract

The sigma 1 receptor (S1R) is a unique transmembrane protein that has been shown to regulate neuronal differentiation and cellular survival. It is expressed within several cell types throughout the nervous system and visceral organs, including neurons and glia within the eye. S1R ligands are therapeutic targets for diseases ranging from neurodegenerative conditions to neoplastic disorders. However, effects of S1R activation and inhibition within glia cells are not well characterized. Within the eye, the astrocytes at the optic nerve head are crucial to the health and survival of the neurons that send visual information to the brain. In this study, we used the S1R-specific agonist, (+)-pentazocine, to evaluate S1R activation within optic nerve head-derived astrocytes (ONHAs). Treatment of ONHAs with (+)-pentazocine attenuated the level and duration of stress-induced ERK phosphorylation following oxidative stress exposure and promoted survival of ONHAs. These effects were specific to S1R activation because they were not observed in ONHAs that were depleted of S1R using siRNA-mediated knockdown. Collectively, our results suggest that S1R activation suppresses ERK1/2 phosphorylation and protects ONHAs from oxidative stress-induced death.

## Introduction

Sigma 1 receptor (S1R) is a small (25kDa), transmembrane protein that is highly conserved but has no known homology to existing mammalian proteins[1]. It is expressed throughout organs and cell types of the central and peripheral nervous, immune, endocrine and reproductive systems[2–5]. Previous studies indicate that S1R functions as a “pluripotent modulator” that interacts with several ion channels and signaling pathways including calcium channels, inositol phosphates, and protein kinases[6]. Despite extensive research, understanding of the molecular cascades triggered by S1Rs is rudimentary. However, the effects of several agonists and antagonists of S1R have been well characterized within models of neurodegenerative disease and cancer therapeutics[7–12]. Overall, these studies indicate that agonists of S1R are pro-survival whereas antagonists of S1R inhibit tumor cell proliferation and induce apoptosis[13]. Therefore, published findings suggest possible applications of sigma ligands in diverse fields including oncology, and treatment of neurodegenerative diseases.

Neuronal and glial cell types throughout the retina and optic nerve contain S1R, and its agonists display robust neuroprotective properties both in vitro and in vivo[7, 14–17]. Recent studies indicate that S1R agonists may offer a treatment option for degenerative eye diseases including photoreceptor dystrophies, and glaucoma[18–21]. Conversely, S1R inhibition is a potential therapeutic option for neoplastic disorders including cancer of the breast and prostate[22, 23]. Understanding the molecular mechanisms that underlie S1R activity is critical to establishing better treatments for eye diseases. Furthermore, evaluation of S1R within ocular tissues contributes to knowledge of how ligands for this receptor would affect the eye, if used systemically as either anti-neoplastic or neuroprotective agents.

A common link between S1R and cell survival mechanisms is the extracellular signal-regulated protein kinase (ERK1/2) cascade. The ERK1/2 molecular signaling pathway regulates basic cell functions, including proliferation, differentiation and survival[24]. Several analyses indicate that S1R interacts with the ERK1/2 pathway, but the mechanism and intracellular consequences of this interaction are not well characterized[20, 21, 25–27]. For example, studies show that the prototypic S1R agonist, (+)-pentazocine (PTZ), protects retinal ganglion cells (RGCs) from hypoxia-induced cell death by increasing activation of ERK1/2[21]. In addition, our recent work describes enhancement of retinal ERK1/2 phosphorylation concomitant with robust protection from excitotoxic injury within retinas of (+)-pentazocine-treated mice[20]. These recent results are in contrast with findings of (+)-pentazocine-mediated decreased ERK1/2 activation within primary microglia cell cultures derived from retina[28]. These findings raise the possibility that the effect of S1R activation on the status of the ERK1/2 pathway might be dependent on the cell type.

S1R agonists, including (+)-pentazocine, have been shown to protect retinal ganglion cells (RGCs) from death under conditions of metabolic and oxidative stress[14, 21]. These protective properties make (+)-pentazocine a potential treatment for optic neuropathies, in which vision loss is caused by degeneration of RGCs[29]. In glaucoma, the most common optic neuropathy, the optic nerve head (ONH) is considered the initial site of disease[30, 31]. It is the most anterior portion of the optic nerve, and the site where RGC axons converge as they leave the eye and pass into the brain. The optic nerve head astrocytes (ONHAs) are the most common glial cells within the ONH. They are vital to the health of the RGCs. Thus, evaluating their response to S1R activation and inhibition is critical to understanding ophthalmic treatment effects of S1R agonists and antagonists.

In the present study, we examined the effects of S1R activation and inhibition in primary cultures of ONHAs. We induced S1R activation using the S1R-specific agonist, (+)-pentazocine. Treatment with this compound attenuated ERK phosphorylation and protected ONHAs from oxidative stress-induced death. In addition, inhibition of S1R using siRNA-mediated knockdown blocked both (+)-pentazocine-induced prosurvival effects, and (+)-pentazocine-induced effects on ERK1/2 activation. Our results therefore suggest that S1R activation protects ONHAs via a mechanism that likely involves modulation of the ERK1/2 signaling pathway.

## Methods and materials

### Primary rat optic nerve head astrocytes (ONHAs) culture

Experiments requiring animals adhered to the ARVO Statement for the Use of Animals in Ophthalmic and Vision Research. Our animal protocol is approved by Augusta University Institutional Animal Care and Use Committee (IACUC, 2011–0338). Primary rat optic nerve head astrocytes were isolated from optic nerve head of Sprague-Dawley (SD) rats (ENVIGO Laboratories, Indianapolis, IN) according to a protocol modified from Mandal et al. (2009) and Beckel et al. (2014)[32, 33]. Briefly, rat pups were euthanized by direct decapitation at postnatal day 3–5. One litter of pups (8–12 animals) was used for each cell culture experiment. Optic nerve head tissue was dissected proximal to the sclera and was digested for 15 minutes using 0.25% trypsin (Invitrogen, Carlsbad, CA) at 37°C. Cells were washed once with ONHAs growth media (Dulbecco’s modified Eagle’s medium DMEM/F12 (Invitrogen, Carlsbad, CA) containing 10% of fetal bovine serum (FBS) (Atlanta Biologicals, Atlanta, GA), 1% penicillin/streptomycin (Invitrogen, Carlsbad, CA), 1% Glutamax (Invitrogen, Carlsbad, CA) and 25ng/ml epidermal growth factor (EGF) (Sigma, St. Louis, MO)), and spun for 5 minutes at 1500rpm. Cells were re-suspended in ONHAs growth media and plated on 0.2% gelatin (Sigma, St. Louis, MO) coated T75 cell-culture flasks. Cells were maintained in a humidified incubator containing 5% CO_2_ at 37°C. Cells were passaged after 7–10 days and were used at passages 2–5. Immunocytochemistry and western blot analyses were performed to identify the purity of ONHA cultures.

### HeLa cell culture

Human cervical adenocarcinoma (HeLa) cells were obtained from ATCC (Manassas, VA. Cat. number # ATCC CCL-2) and maintained in a humidified incubator containing 5% CO_2_ at 37°C. HeLa cells were cultured in Dulbecco’s modified Eagle’s medium (DMEM) (Invitrogen, Carlsbad, CA) supplemented with 10% FBS and 1% penicillin/streptomycin.

### Immunocytochemistry

Cells were fixed with 4% paraformaldehyde (Electron Microscopy Sciences, Hatfield, PA) at room temperature for 15 min, followed by washing with PBS three times. Cells were then membrane-permeabilized with 0.3% triton X-100 in PBS at room temperature for 10 min. Next, cells were blocked with 0.1% triton X-100 in PBS (PBST) with 10% goat serum (Sigma, St. Louis, MO) at room temperature for 1 hour, then incubated in primary antibody (Glial Fibrillary Acidic Protein (GFAP) 1:500, Dako, Carpinteria, CA; Neural Cell Adhesion Molecule (NCAM) 1:100, Santa Cruz, Dallas, TX; Iba-1 1:500, Wako, Richmond, VA; Oligodendrocyte Specific Protein (OSP) 1:500, Abcam, Cambridge, MA; S1R 1:500) at 4°C overnight. The S1R rabbit polyclonal antibody was raised from peptide sequence SEVYYPGETVVHGPGEATDVEWG (corresponds to residues 143–165 of rat S1R), and generated within the laboratory of Dr. Sylvia Smith. This antibody has been used in numerous publications since 2002 as a tool for study of S1R expression and function[7, 17, 34]. The second day, cells were incubated in secondary antibody (Alexa Fluor 555-labeled goat anti rabbit 1:1000; Alexa Fluor 488-labeled goat anti mouse 1:1000, Invitrogen, Carlsbad, CA) at room temperature for 1 hour. After washing 3 times with PBST, coverslips were mounted with Fluoroshield with DAPI (Sigma, St. Louis, MO). Cells were observed for immunofluorescence using a Zeiss Axioplan-2 microscope (Carl Zeiss, Oberkochen, Germany) equipped with AxioVision program (version 4.6.3) and a high-resolution microscopy camera.

### Western blot analysis

Rat optic nerve head astrocytes were lysed in RIPA buffer (Sigma, St. Louis, MO) containing 2mM sodium orthovanadate and 1% protease inhibitor cocktail (Thermo Scientific, Waltham, MA). Cell lysates were centrifuged at 14,000g for 30 minutes. Protein concentration was measured by Bradford assay (Bio-Rad, Hercules, CA). Proteins were separated by electrophoresis on a 10% SDS-polyacrylamide gel, then transferred to a nitrocellulose membrane (Thermo Scientific, Waltham, MA). The membrane was blocked with 5% nonfat milk in Tris-buffered saline-0.05%Tween 20 (TBST) for 1 hour at room temperature, then incubated overnight at 4°C with primary antibodies (GFAP 1:1000; Iba-1 1:500; OSP 1:1000; S1R 1:1000; p-ERK 1:500; ERK 1:500; glyceraldehyde-3-phosphate dehydrogenase (GAPDH) 1:2000). After three washes in TBST, the membrane was incubated for 1 hour with an appropriate Horseradish Peroxidase (HRP)-conjugated secondary antibody at room temperature. Proteins were visualized by incubating with a SuperSignal West Pico chemiluminescent substrate (Thermo Scientific, Waltham, MA) and quantified by densitometry with ImageJ software (http://imagej.nih.gov/ij/; provided in the public domain by the National Institutes of Health, Bethesda, MD). Blots were stripped and re-probed for loading controls. GFAP antibody was purchased from Dako, Carpinteria, CA; Iba-1 antibody from Wako, Richmond, VA; OSP antibody from Abcam, Cambridge, MA; phospho-ERK, ERK polyclonal antibodies from Cell Signaling Technology, Danvers, MA; GAPDH monoclonal antibody, HRP-conjugated anti-rabbit IgG, and HRP-conjugated anti-mouse IgG from Santa Cruz Biotechnology, Dallas, TX.

### Cell culture treatment

For MTT assay, ONHAs were seeded onto 96-well plates (10,000 cells/well). To determine (+)-pentazocine (Sigma, St. Louis, MO) toxicity to ONHA, ONHAs were exposed to increasing (+)-pentazocine concentrations (1, 3, 10, 50μM) for 24 hours. To determine H_2_O_2_ (Sigma, St. Louis, MO) toxicity to ONHA, ONHA were exposed to increasing H_2_O_2_ concentrations (50, 100, 150, 200, 250, 500μM) for 24 hours. To detect if (+)-pentazocine would protect ONHAs from H_2_O_2_ induced cell death, ONHAs were pre-treated with 10μM (+)-pentazocine for 1 hour followed by 100 μM H_2_O_2_ cotreatment with 10μM (+)-pentazocine for 24 hours. To detect if ERK inhibitor U0126 (Cell Signaling Technology, Danvers, MA) would protect ONHAs from H_2_O_2_ induced cell death, ONHAs were pre-treated with 10μM U0126 for 1 hour followed by 100 μM H_2_O_2_ cotreatment with 10μM U0126 for 24 hours.

To detect changes in phosphorylated ERK levels in ONHAs exposed to H_2_O_2_ and (+)-pentazocine, ONHAs were seeded onto 24 well plates at 20,000 cells /well. Cells were treated with 100μM H_2_O_2_ with or without 1-hour pre-treatment of 10μM (+)-pentazocine. After 15 minutes, 30 minutes, 1hour, 3 hours and 24 hours, cell lysates were collected for western blot to detect p-ERK, total ERK and GAPDH expression.

### MTT assay

Rat optic nerve head astrocytes were seeded onto 96-well plates at a density of 10,000 cells/well in ONHAs growth media. After incubation with H_2_O_2_ with or without (+)-pentazocine for 24 hours, medium was removed and MTT (1.2mM in culture medium) (Invitrogen, Carlsbad, CA) was added and incubated for 4h at 37°C. Solubilization with dimethyl sulfoxide (DMSO) (Sigma, St. Louis, MO) was performed according to protocol directions. Absorbance was measured at 540nm using a microplate reader (VERSA max, Molecular Devices, Sunnyvale, CA). Experiments were performed in quadruplicate and repeated 3 times.

### Reactive oxygen species (ROS) detection

For intracellular ROS detection, ONHAs grown on coverslips were washed with PBS and incubated with 5μM CellROX Green reagent (Invitrogen, Carlsbad, CA) in the dark for 30 min at 37°C. After washing and fixing with 4% of paraformaldehyde, cells were mounted with Fluoroshield with DAPI. Intracellular levels of ROS corresponding to emitted fluorescence were observed by Zeiss Axioplan-2 microscope (Carl Zeiss, Oberkochen, Germany) equipped with AxioVision program (version 4.6.3) and a high-resolution microscopy camera.

### SiRNA transfection

S1R siRNA and non-targeting, negative control siRNA (scrambled siRNA) were purchased from Dharmacon (Lafayette, CO, USA). S1R or scrambled siRNA were transfected into HeLa cells and rat primary ONHAs using Lipofectamine RNAiMAX transfection reagent (Invitrogen, Carlsbad, CA) following the manufacturer's protocol. Briefly, the night before transfection, cells were plated 20,000/ well onto 24 well plates or 5000/well onto 96 well plates. For transfection, 10pmol S1R siRNA or scrambled siRNA were mixed with RNAiMax reagent for 5 minutes at room temperature. Then RNA-lipid complexes were added to cells. For confirmation of S1R knockdown, cells were harvested 3 days and 6 days post-transfection, and lysed in RIPA buffer. Lysates were assayed for S1R expression using western blot. Human S1R siRNA target sequences: GAAUGCGGGUGGCUGGAUG; GGCUUGAGCUCACCACCUA; GAGCUGGCCUUCUCUCGUC; CCAAACACAUGGAUGGUGG. Rat S1R siRNA target sequences: GAACACAUACCACGAGCUU; GUGCACACCUUGUCGGUCU; GUGAGGAAGGACAUACAUA; AGACUUGUAUGUACUGUAA. Scrambled siRNA sequence: UGGUUUACAUGUCGACUAA.

### Statistical analysis

Data for MTT assay, ROS detection, and western blot were analyzed using t-test, one- or two-way ANOVA followed by Tukey-Kramer post hoc test for multiple comparisons. Significance was set at P<0.05 (Prism; GraphPad Software, Inc. La Jolla, CA).

## Results

### Culture of ONHA and expression of S1R

**Fig 1:**
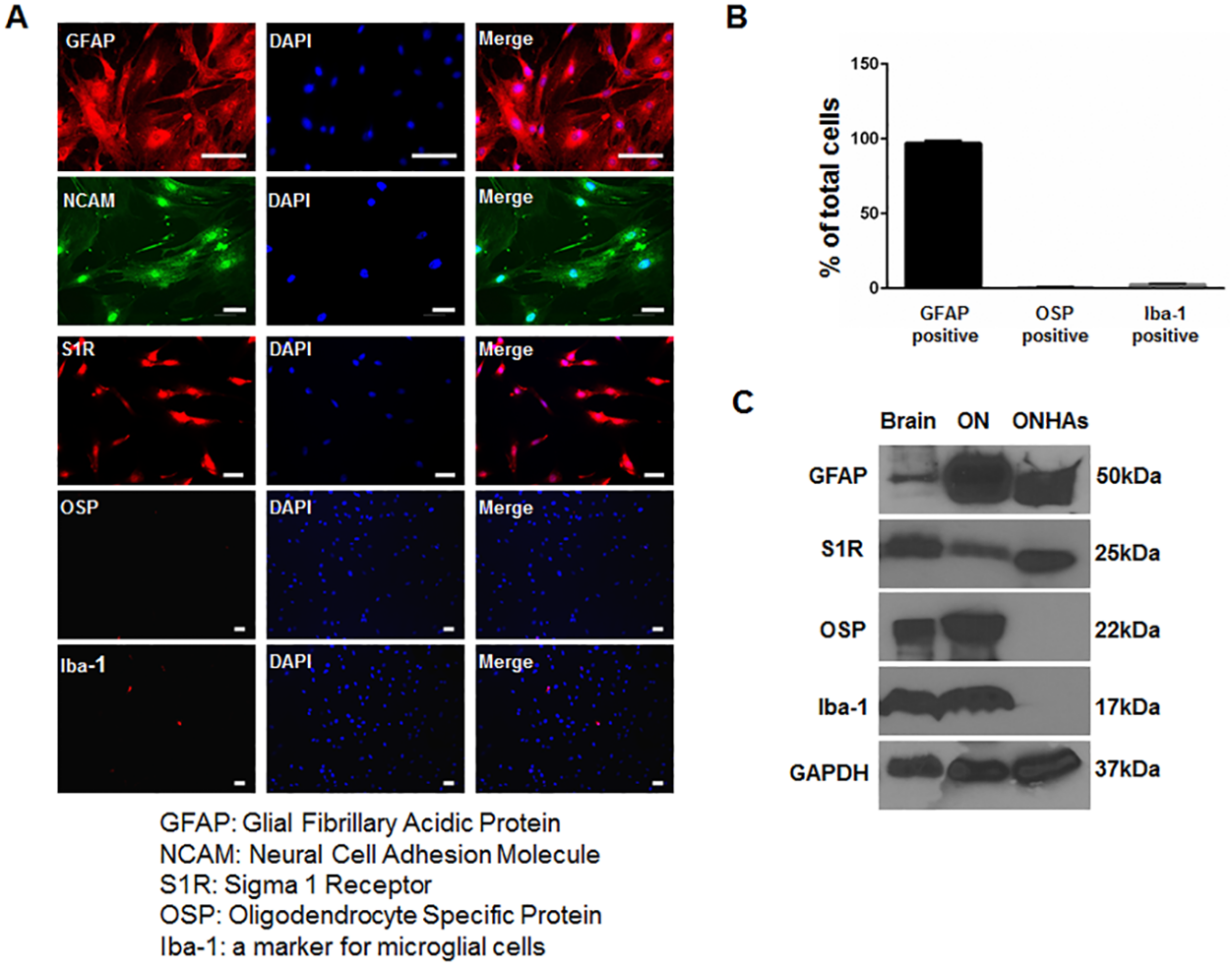
Characterization of cultured primary rat optic nerve head astrocytes (ONHA). (A) ONHAs were fixed and probed with antibodies against GFAP (red) and NCAM (green), S1R (red), OSP (red), and Iba-1 (red). The cells were counterstained with DAPI to label DNA (blue) as a marker for nuclei. Scale bar: 50μm. (B) Quantitative analysis shows that more than 95% of the cells in culture express GFAP. (C) The cell lysates from ONHAs (lane 3) were positive for GFAP, a marker for astrocytes and S1R, but negative for Iba-1, a marker for microglial cells, and OSP, a marker for oligodendrocytes. The protein extract from rat brain (lane 1) and from rat optic nerve tissue (lane 2) were used as positive controls.

Previous work indicated that S1R is expressed within the RGCs and glial cells of the optic nerve, but it has not been evaluated in ONHAs [16]. We cultured ONHAs from rats between postnatal day 3 and 5 using previously described methods[32, 33]. We used rats for these analyses because many mechanistic studies involving S1R have utilized rodents. In addition, due to its relative size, the optic nerve head of the rat is more easily isolated from surrounding tissues than that of the mouse. Our cultured cells expressed S1R, GFAP and NCAM, but did not express markers for oligodendrocytes or microglia (Fig 1).

### Protection of ONHAs from oxidative stress-induced cell death by the S1R agonist, (+)-pentazocine

**Fig 2:**
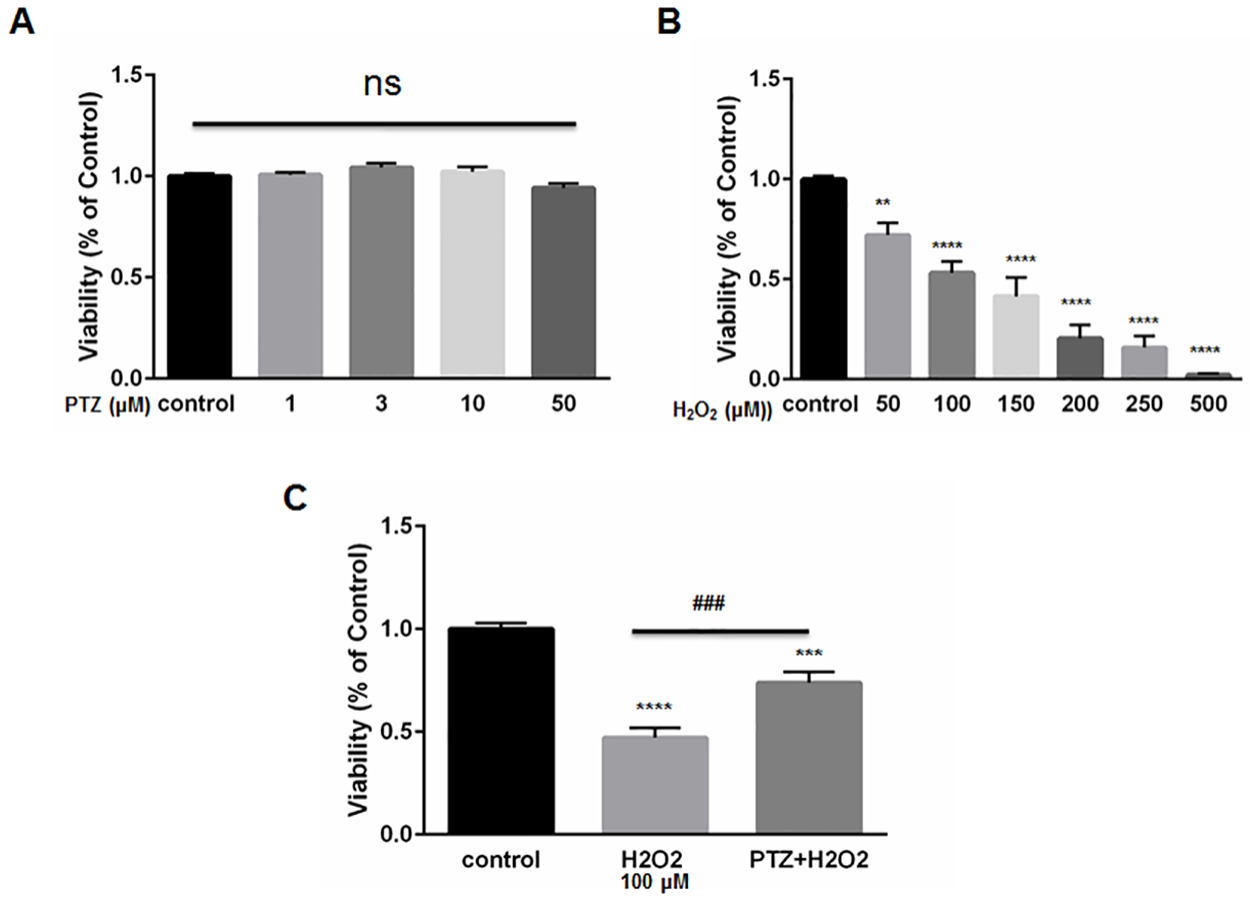
The effect of (+)-pentazocine and H_2_O_2_ on ONHAs viability. (A) ONHAs were treated with (+)-pentazocine (PTZ) at varying concentrations (1,3,10 or 50μM) for 24 hours. MTT assay was performed to assess viability. Treatment with PTZ did not cause a significant change in percentage viability compared to the untreated control cells. At the highest concentration of PTZ (50μM), there was a trend toward decrease in viability that was not significant. (B) ONHAs were exposed to various H_2_O_2_ concentrations (50,100,150, 200, 250, 500μM) for 24 hours. H_2_O_2_ induced ONHA death in a dose dependent manner. (C) ONHAs were treated with 100μM H_2_O_2_ for 24 hours in the presence or absence of PTZ (10μM, 1 hour pretreatment followed by co-treatment). Exposure to H_2_O_2_ significantly decreased viability compared to non-exposed cells. Compared to H_2_O_2_-exposure with no PTZ treatment, the H_2_O_2_-exposed, PTZ-treated ONHAs showed significantly increased viability. Data were analyzed using one-way ANOVA followed by Tukey-Kramer post hoc test for multiple comparisons. Significantly different from control *P<0.05, **P<0.01, ***P<0.001, ****P<0.0001. Significantly different between groups ###P<0.001. Experiments were performed in quadruplicate and repeated 3 times.

We exposed ONHAs to increasing doses of (+)-pentazocine. Cell viability was then measured using the MTT assay. (+)-Pentazocine did not cause a significant change in percentage viability compare to the untreated control (Fig 2A). This result indicates that the S1R agonist, (+)-pentazocine, is minimally toxic to ONHA. We then exposed ONHA cultures to progressively increasing levels of oxidative stress by applying increasing concentrations of the chemical oxidant, hydrogen peroxide (H_2_O_2_). Results showed a concentration-dependent decrease in cell viability when cultures were exposed to increasing levels of oxidative stress (Fig 2B). The ONHAs were then subjected to death-inducing levels of H_2_O_2_ while undergoing treatment with the S1R agonist, (+)-pentazocine. We chose to expose cells to a 10μM concentration of (+)-pentazocine because previous studies indicate that this dosage is neuroprotective of retinal ganglion cells in vitro [21]. In addition, this concentration of (+)-pentazocine has been shown to attenuate oxidative stress in retinal Muller glial cells [35]. When cell cultures received (+)-pentazocine for one hour prior to and during the H_2_O_2_ application, the oxidative stress-induced cell death was significantly mitigated (Fig 2C).

### S1R agonist, (+)-pentazocine inhibits ROS generation induced by oxidative stress in ONHAs

**Fig 3:**
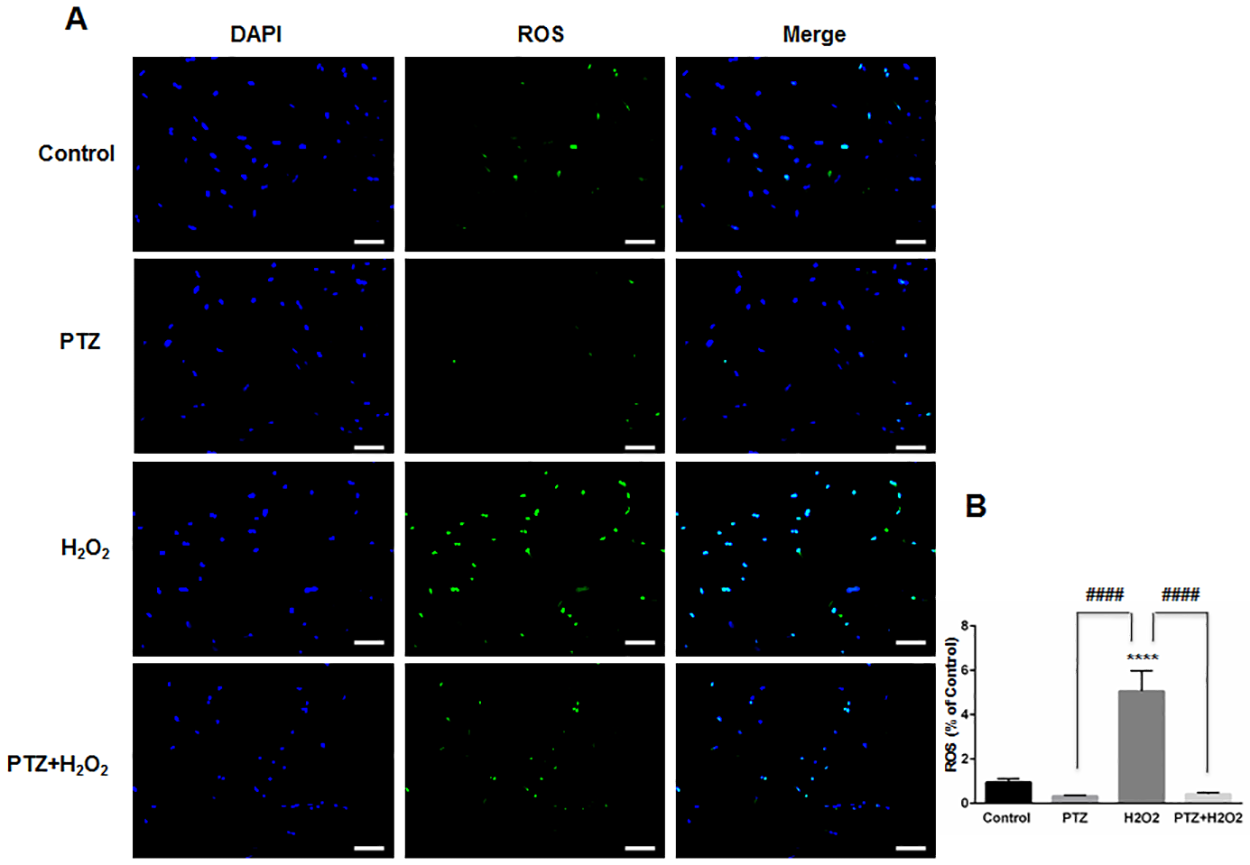
Effect of PTZ on ROS generation when ONHAs exposed to H_2_O_2_. (A) Representative images of ONHAs treated with 100μM H_2_O_2_ for 24 hours in the presence or absence of PTZ (10μM, 1 hour pretreatment followed by co-treatment). ROS generation was visualized using CellROX Green reagent. Scale bar: 100μm. (B) Quantitative analysis of intracellular ROS. For each group, three coverslips were quantified, and eight images were taken from each coverslip. Mean signal intensity was quantified by ImageJ. Intracellular ROS generation increased when ONHAs were exposed to H_2_O_2_. The ROS generation was inhibited by PTZ. Data were analyzed using one-way ANOVA followed by Tukey-Kramer post hoc test for multiple comparisons. Significantly different from control ****P<0.0001. Significantly different between groups ####P<0.0001. Experiments were repeated 3 times.

Studies indicate that increased intracellular reactive oxygen species (ROS) will affect the functional capacity of astrocytes, independent of their viability[36]. For example, an increase in intracellular ROS leads to altered astrocytic glutamate uptake and impaired glioprotection of associated neurons[37]. Therefore, we measured intracellular ROS generation within ONHAs, with and without (+)-pentazocine, in the presence of oxidative stress. Intracellular ROS detection was performed using CellROX Green Reagent. We found that exposure of ONHAs to H_2_O_2_ caused a significant increase in intracellular ROS (Fig 3). When the oxidative stress-exposed cell cultures were treated with (+)-pentazocine, intracellular ROS generation was significantly suppressed (Fig 3). Treating control ONHAs with (+)-pentazocine in the absence of oxidative stress caused no significant change in intracellular ROS generation (Fig 3).

### (+)-Pentazocine-mediated effects on ERK1/2 activation in oxidative stress-exposed ONHAs

**Fig 4:**
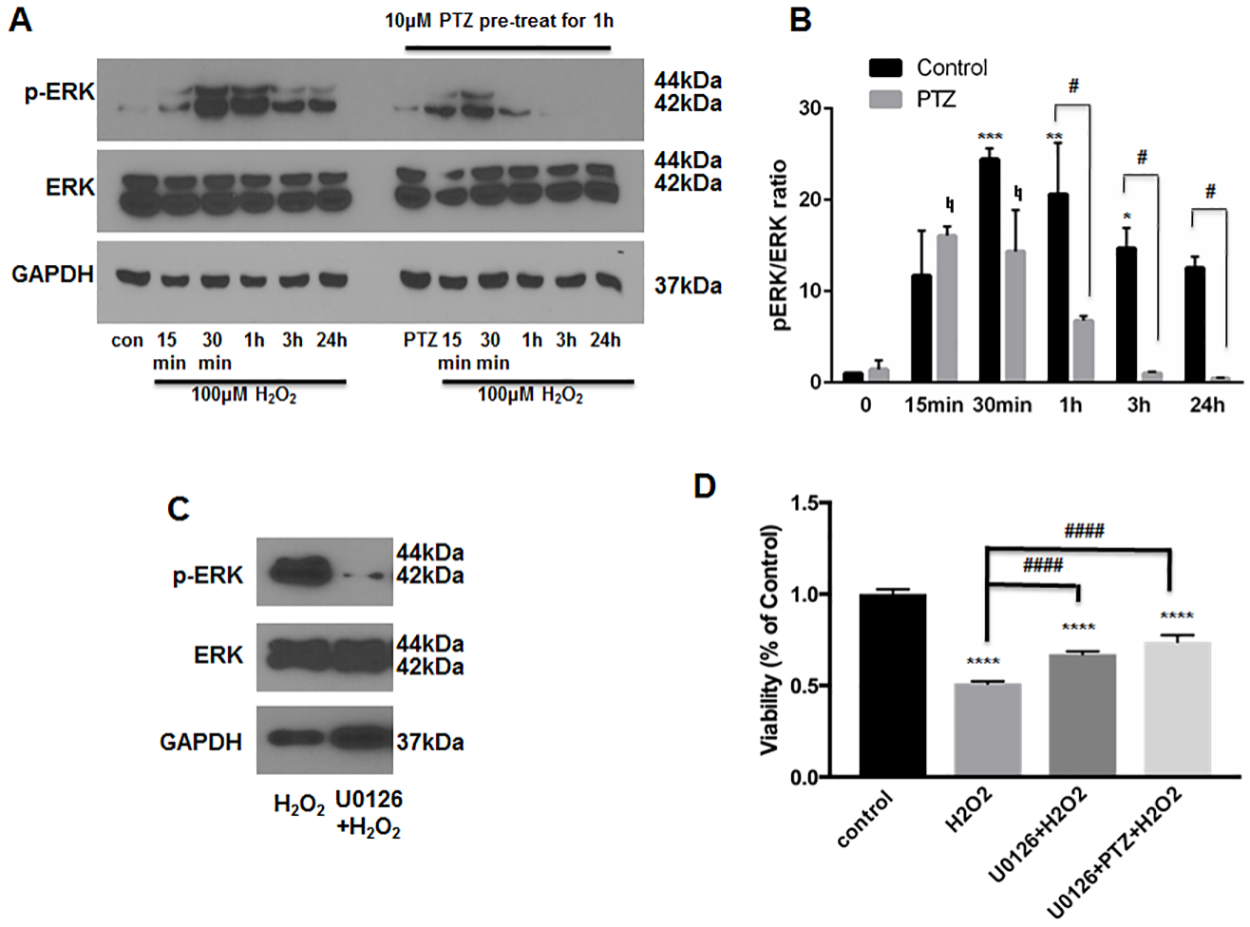
Inhibition of ERK1/2 phosphorylation in ONHAs. (A) ONHAs were incubated with 100μM H_2_O_2_ at 37°C for 15 minutes, 30 minutes, 1 hour, 3 hours and 24 hours in the presence or absence of PTZ (10μM, 1 hour pretreatment followed by cotreatment). Western blot analysis of cell lysates is shown. Phosphorylation of ERK was increased at the 15 minute time point following H_2_O_2_ application, and peaked between 30 minutes and 1 hour. Lysates derived from H_2_O_2_-exposed cells treated with PTZ showed decreased ERK phosphorylation at 1 hour, 3 hour and 24 hour time points. (B) Quantitative analysis of pERK levels represented as pERK normalized to total ERK. Results are presented as fold change of the pERK/total ERK ratio derived from H_2_O_2_ exposed cells versus non-H_2_O_2_ exposed cells. Experiments were repeated 3 times. Data were analyzed using two-way ANOVA followed by Tukey-Kramer post hoc test for multiple comparisons. Significance levels for pERK change in the untreated (no PTZ) control group (black columns) are as follows: *p<0.05 **p<0.01, ***p<0.001. Significance level for pERK change in the PTZ-treated group (gray columns) is as follows: **♮**p<0.05. Significance between the untreated control group (black columns) and PTZ-treated group (gray columns) at the 1 hour, 3 hour and 24 hour time points is represented as follows: #p<0.05 Note that the change in pERK between these groups was not significant at the 0 minute, 15 minute, and 30 minute time points. (C) ONHAs were exposed to 100μM H_2_O_2_ for 24 hours in the presence or absence of U0126 (10μM, 1hour pretreatment followed by co-treatment). Western blot showed decreased ERK phosphorylation levels under conditions of U0126 treatment compared with no treatment. (D) ONHAs were treated with 100μM H_2_O_2_ for 24 hours in the presence or absence of U0126 (10μM, 1hour pretreatment followed by co-treatment). MTT assay was performed to assess viability. Exposure to H_2_O_2_ significantly decreased ONHA viability and treatment with U0126 increased viability compared to the non-treated, H_2_O_2_-exposed cells. Combined treatment with PTZ and U0126 did not show a significant additive effect on cell viability. Data were analyzed using one-way ANOVA followed by Tukey-Kramer post hoc test for multiple comparisons. Significantly different from control: ****p<0.0001. Significantly different between groups: ####p<0.0001. Experiments were performed in quadruplicate and repeated 3 times.

Several recent studies have linked the pro-survival effects of S1R agonists to changes in ERK1/2 phosphorylation status[20, 21, 26]. For example, ERK activation, induced by stimulation of S1R, leads to protection of purified RGCs under conditions of oxygen-glucose deprivation[21]. To examine the effect of (+)-pentazocine on ERK1/2 activation within ONHAs, we first analyzed ERK1/2 phosphorylation within ONHA cultures exposed to H_2_O_2_ (100μm) at a concentration previously shown to cause severe cellular stress (Fig 2C). Cultures were treated with H_2_O_2_ (100*μ*M) for 15 minutes, 30 minutes, 1 hour, 3 hours, or 24 hours. The cells were then harvested and western blot analysis was performed to assay for the phosphorylated fraction of total ERK1/2 protein (pERK1/2). Consistent with previous studies of oxidative stress-exposed astrocytes, we found that exposure of ONHAs to H_2_O_2_ led to increased levels of pERK1/2 at time points of 15 minutes to 24 hours following application, with peak activation occurring at the 30 minute time point (Fig 4A)[38]. Cultures were then treated with (+)-pentazocine for one hour prior to and during the H_2_O_2_ exposure. Resultant analyses showed that the level and duration of ERK1/2 phosphorylation within (+)-pentazocine-treated cells was reduced in comparison to the control (Fig 4A and 4B). In addition, the bell-curve of ERK1/2 phosphorylation was shifted to earlier time points. In contrast to control cells, peak phosphorylation of ERK1/2 was observed as early as the 15 minute time point in pentazocine-treated cells (Fig 4A and 4B). Despite this temporal shift, the total level of ERK1/2 phosphorylation at time points beyond 30 minutes was significantly reduced in pentazocine-treated cells in comparison to the control (Fig 4A and 4B).

### Inhibition of ERK1/2 phosphorylation is pro-survival in ONHAs

As described above, treatment of oxidative stress-exposed ONHAs with (+)-pentazocine lead to increased survival and attenuation of ERK1/2 activation. To determine whether decreased ERK1/2 phosphorylation could be a mechanism through which (+)-pentazocine promotes survival, we analyzed the effects of inhibiting ERK1/2 activation in ONHAs. To do this, we treated ONHA cultures with U0126, an inhibitor of ERK phosphorylation, for one hour prior to and during application of oxidative stress. Viability of the U0126-treated ONHAs was assayed after 24 hours of exposure to H_2_O_2_. Cell cultures treated with U0126 showed reduced cell death and ERK1/2 phosphorylation compared with untreated cultures (Fig 4C and 4D). These results suggest that inhibition of ERK1/2 activation promotes survival of ONHAs under conditions of oxidative stress.

### Knockdown of S1R within ONHAs blocks (+)-pentazocine-mediated pro-survival effects

Results presented in Fig 2. suggest that the S1R specific agonist, (+)-pentazocine, protects ONHAs from oxidative stress-induced cell death. To test the hypothesis that (+)-pentazocine-mediated cellular survival occurs through an S1R-dependent mechanism, we depleted S1R within ONHAs using small interfering (si)RNAs.

**Fig 5:**
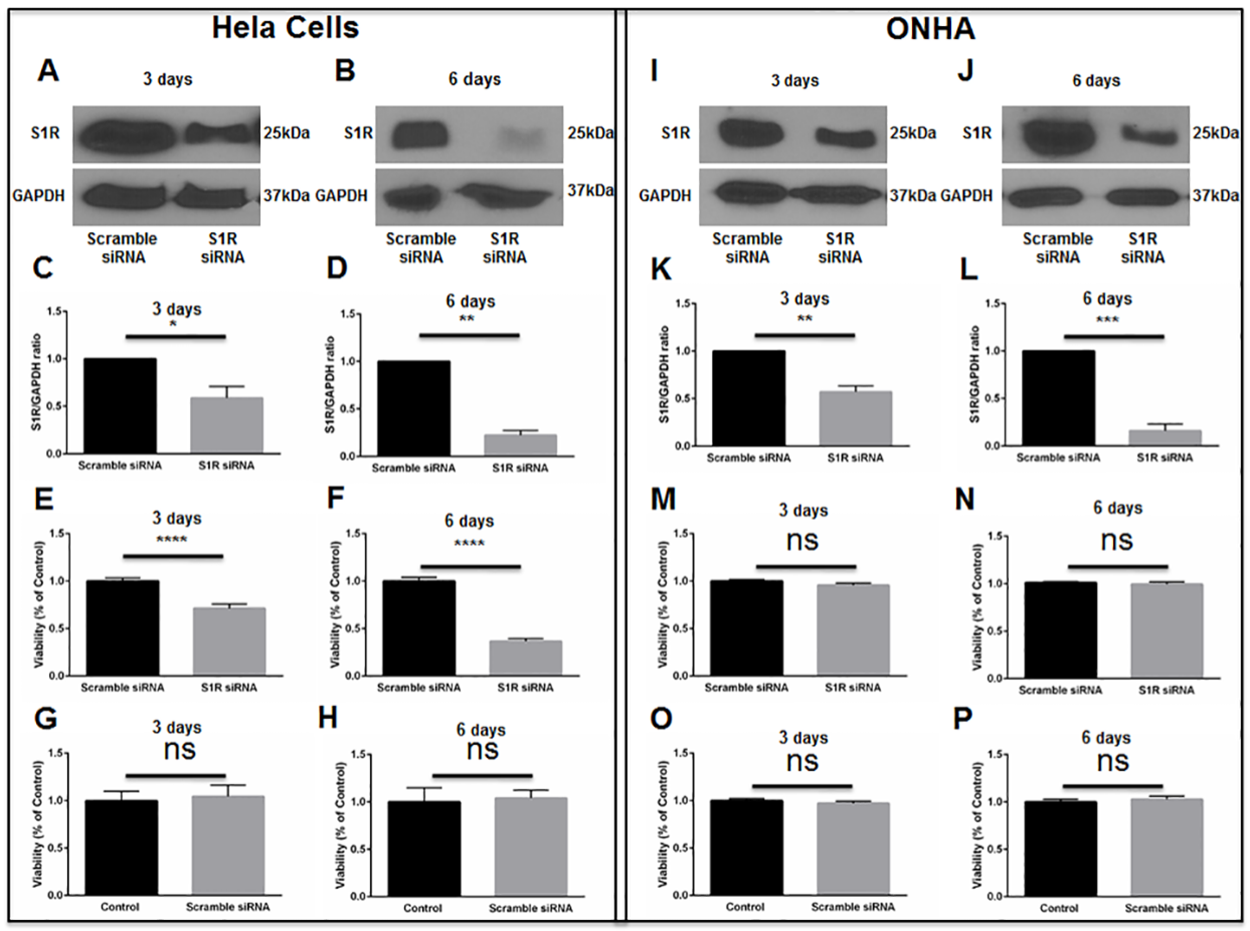
S1R knockdown in HeLa cells and ONHAs. HeLa cells were transfected with human scrambled siRNA or with human S1R siRNA. Western blot analysis of S1R levels at 3 days (A) and 6 days (B) following transfection is shown. Quantitation of western blot results shows S1R levels normalized to GAPDH as the internal control (C, D). Results are presented as fold change of S1R levels derived from the S1R siRNA-transfected cells compared to S1R levels derived from scrambled siRNA-transfected cells. Data were analyzed using t-test. Significantly different from control *p<0.05, **p<0.01. Experiments were repeated 3 times. MTT assay was performed to assess viability at 3 days (E) and 6 days (F) after scrambled or S1R siRNA transfection. Viability was significantly decreased in S1R siRNA-transfected HeLa cells compared to scrambled siRNA-transfected cells. Transfection with scrambled siRNA did not cause significant HeLa cell death compared to non-transfection control (G, H). Data were analyzed using t-test. Significantly different from control ****p<0.0001. Experiments were performed in quadruplicate and repeated 3 times. ONHAs were transfected with rat scrambled siRNA or with rat S1R siRNA. Western blot analysis of S1R levels at 3 days (I) and 6 days (J) following transfection is shown. Quantitation of western blot results shows S1R levels normalized to GAPDH as the internal control (K, L). Results are presented as fold change of S1R levels derived from the S1R siRNA-transfected cells compared to S1R levels derived from scrambled siRNA-transfected cells. Data were analyzed using t-test. Significantly different from control: **p<0.01, ***p<0.001. Experiments were repeated 3 times. MTT assay was performed to assess viability at 3 days (M) and 6 days (N) after scrambled or S1R siRNA transfection. Viability was not significantly changed in S1R siRNA-transfected ONHAs compared to scrambled siRNA-transfected cells. Transfection with scrambled siRNA did not cause significant ONHA death compared to non-transfection control (O, P). Experiments were performed in quadruplicate and repeated 3 times.

Initial knockdown experiments were performed using a human cervical cancer cell line (HeLa cells), because preliminary conditions for transfection were more easily determined using a cell line versus primary ONHAs. Cell cultures were transfected with scrambled siRNA or with S1R siRNA targeting human S1R. Western blot analysis was used to measure the level of S1R protein at 3 days and 6 days following transfection. These assays showed no change in the level of S1R protein in scrambled siRNA-transfected cultures. However, at 3 days and 6 days following transfection with S1R siRNA, we found significant decreases in S1R protein levels (Fig 5A–5D). Using MTT assay, we then measured HeLa cell viability at time points of 3 days and 6 days following siRNA transfection. Cells transfected with scrambled siRNA showed no change in viability compared with non-transfected cultures (Fig 5G and 5H). However, knockdown of S1R resulted in significant baseline HeLa cell death, without exposure to oxidative stress (Fig 5E and 5F). These results are consistent with previous reports showing that S1R inhibition restricts growth and causes death within rapidly dividing cell cultures[22, 23].

We then performed knockdown of S1R within primary ONHAs. Cultures were transfected with scrambled siRNA or S1R siRNA targeting rat S1R. Consistent with results obtained in HeLa cells, S1R knockdown efficiencies were significant at 3 and 6 days following transfection of ONHAs (Fig 5I–5L). In contrast to results observed in HeLa cells, knockdown of S1R within ONHAs did not significantly affect cellular viability (Fig 5M–5P).

**Fig 6:**
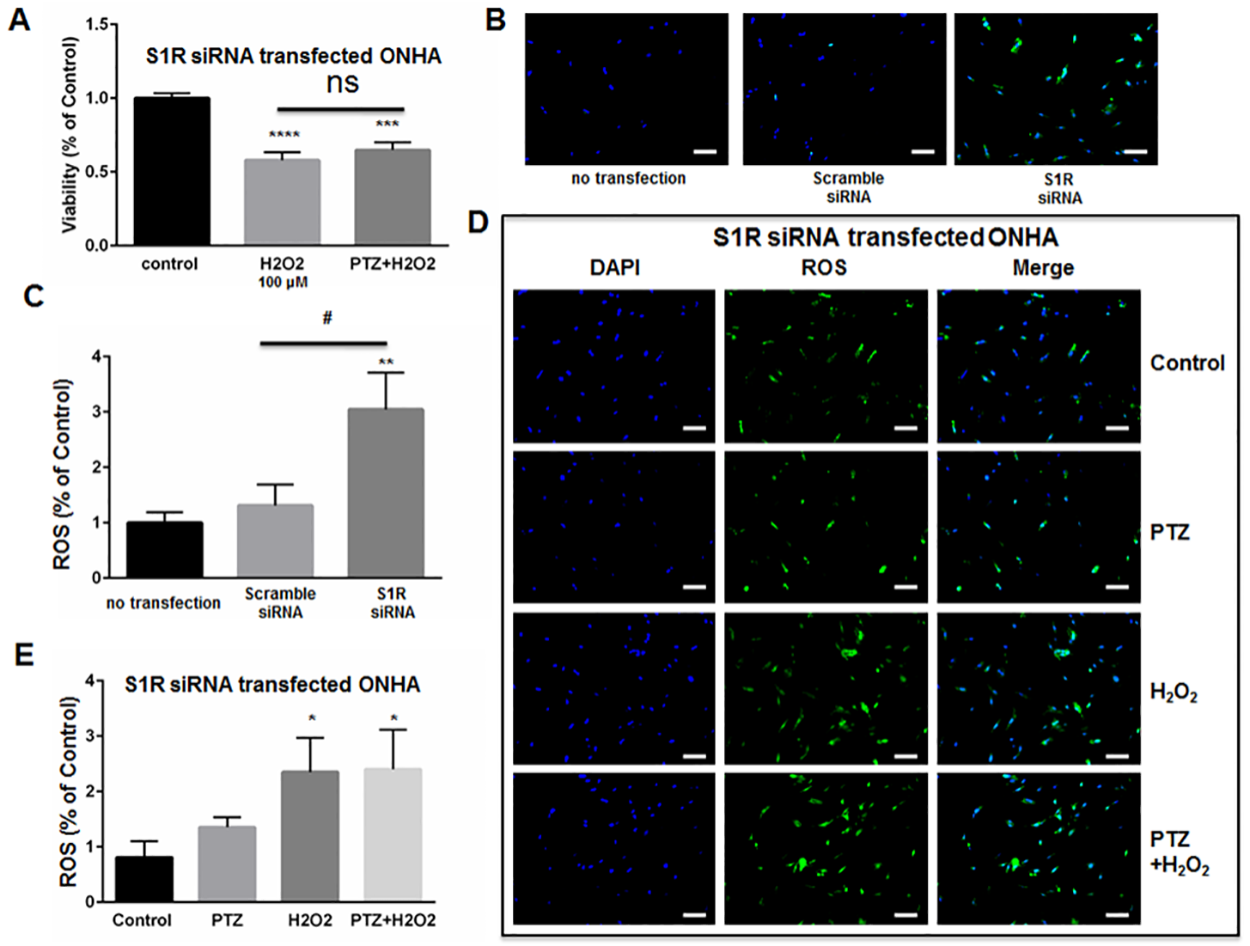
Knockdown of S1R within ONHAs blocks (+)-pentazocine-mediated suppression of ROS generation. (A) Five days following transfection with S1R siRNA, ONHAs were treated with 100μM H_2_O_2_ for 24 hours in the presence or absence of PTZ (10μM, 1 hour pretreatment followed by co-treatment). MTT assay was performed to assess viability.100μM H_2_O_2_ induced 50% cell death, and PTZ treatment did not significantly increase cell viability. (B-C) Effect of S1R knockdown on ROS generation in ONHAs. Data were analyzed using one-way ANOVA followed by Tukey-Kramer post hoc test for multiple comparisons. Significantly different from control: ***p<0.001, ****p<0.0001. (B) Representative images of ONHAs six days following transfection with either scrambled siRNA or S1R siRNA. ROS generation was visualized using CellROX Green reagent. Scale bar: 100μm. (C) Quantitative analysis of intracellular ROS. For each group, three coverslips were quantified, and eight images were taken from each coverslip. Mean signal intensity was quantified by ImageJ. Transfection with scrambled siRNA did not significantly increase ROS generation compared with non-transfected control cells. S1R siRNA-transfected ONHAs showed increased intracelluar ROS compared with scrambled siRNA-transfected ONHAs. Data were analyzed using one-way ANOVA followed by Tukey-Kramer post hoc test for multiple comparisons. Significantly different from control: **p<0.01. Significantly different between groups: #p<0.05. (D) Representative images of S1R siRNA-transfected ONHAs treated with 100μM H_2_O_2_ with or without PTZ for 24 hours. Scale bar: 100μm. (E) Quantitative analysis of intracellular ROS. For each group, three coverslips were quantified, and eight images were taken from each coverslip. Mean signal intensity was quantified by ImageJ. ROS generation increased when cells were incubated with H_2_O_2_. The ROS generation was not inhibited by PTZ treatment. Data were analyzed using one-way ANOVA followed by Tukey-Kramer post hoc test for multiple comparisons. Significantly different from control *p<0.05. Experiments were repeated 3 times.

We next determined whether (+)-pentazocine could protect S1R-knockdown ONHAs from oxidative stress-induced cell death. Therefore, we subjected knockdown cell cultures to 100μM H_2_O_2_ in the presence or absence of (+)-pentazocine treatment (Fig 6A). Non-treated knockdown cultures exposed to 100μM H_2_O_2_ showed significant cell death at 6 days post-transfection with S1R siRNA (Fig 6A). When identically-exposed S1R knockdown cultures were treated with (+)-pentazocine, no significant protection from cell death was observed (Fig 6A).

As described above (Fig 3), we previously observed decreased ROS generation when H_2_O_2_-exposed ONHAs were treated with (+)-pentazocine. To determine whether the (+)-pentazocine-mediated inhibition of ROS generation occurred through S1R, we measured reactive oxygen species (ROS) generation in S1R knockdown ONHAs. Measurements were performed under conditions of H_2_O_2_ exposure, in the presence and absence of (+)-pentazocine. Intracellular ROS levels were assayed as described in Fig 3. In contrast to scrambled siRNA–transfected cells, the S1R-knockdown ONHAs showed high baseline ROS generation (Fig 6B and 6C). Exposure of S1R siRNA-transfected ONHAs to H_2_O_2_ resulted in a significant increase in intracellular ROS generation (Fig 6D and 6E). Treatment of the H_2_O_2_-exposed, S1R-knockdown cultures with (+)-pentazocine did not significantly inhibit ROS generation (Fig 6D and 6E).

Overall, these results suggest that knockdown of S1R blocks (+)-pentazocine-induced mitigation of ROS generation and inhibits (+)-pentazocine-mediated pro-survival effects in oxidative stress-exposed ONHA. Thus, S1R appears to be necessary for the survival-promoting effects of (+)-pentazocine on ONHA.

### Knockdown of S1R within ONHAs increases baseline ERK1/2 phosphorylation and blocks (+)-pentazocine-mediated suppression of ERK1/2 phosphorylation

**Fig 7:**
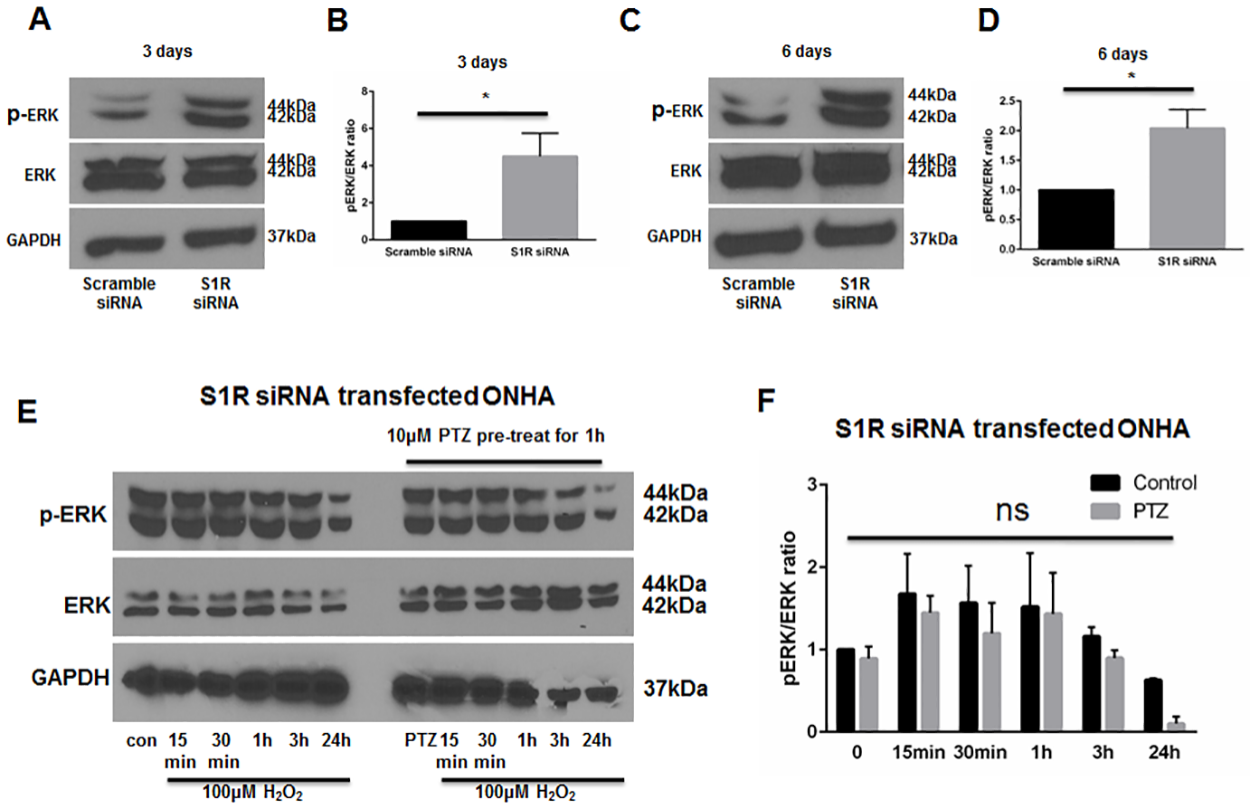
Knockdown of S1R within ONHAs blocks the (+)-pentazocine-mediated suppression of ERK1/2 phosphorylation. Phophorylation of ERK was detected after 3 days (A) and 6 days (C) following transfection of scrambled or S1R siRNA in ONHA. At 3 days (A) and 6 days (C) following S1R siRNA transfection, pERK was increased compared to scrambled siRNA control. (B, D) Quantitative analysis of pERK levels represented as pERK normalized to total ERK. Results are presented as fold change of the pERK/total ERK ratio derived from S1R siRNA transfected cells versus scrambled siRNA transfected control cells. Data were analyzed using t-test. Significantly different from control: *p<0.05. Experiments were repeated 3 times. (E) Effect of PTZ on H_2_O_2_–exposed S1R siRNA transfected ONHA. 5 days after S1R siRNA transfection, ONHA were incubated with 100μM H_2_O_2_ at 37°C for 15 minutes, 30 minutes, 1 hour, 3 hours and 24 hours in the presence or absence of PTZ (10μM, 1 hour pretreatment followed by cotreatment). Western blot analysis is shown. (F) Quantitative analysis of pERK levels represented as pERK normalized to total ERK. Results are presented as fold change of the pERK/total ERK ratio derived from H_2_O_2_ exposed, S1R siRNA transfected cells versus control non-H_2_O_2_ exposed, S1R siRNA transfected cells. Data were analyzed using two-way ANOVA followed by Tukey-Kramer post hoc test for multiple comparisons.

As described above, our results indicate that treatment of ONHAs with (+)-pentazocine inhibits oxidative stress-induced ERK1/2 activation (Fig 4). To address whether suppression of ERK1/2 activation can occur through S1R, we performed knockdown of S1R in ONHAs, followed by western blot analysis of pERK1/2 levels. S1R-knockdown cell cultures were treated with or without oxidative stress (H_2_O_2_), in the presence or absence of (+)-pentazocine. Measurement of ERK1/2 phosphorylation was performed at time points of 15 minutes to 24 hours following application of H_2_O_2_. In contrast to scrambled siRNA-transfected cells, the S1R-knockdown ONHAs showed a high baseline level of pERK (Fig 7A–7D), with a trend toward increased ERK1/2 activation in the presence of oxidative stress. Treatment with (+)-pentazocine did not significantly suppress ERK1/2 phosphorylation (Fig 7E and 7F). Based on these results, we conclude that S1R functions to decrease pERK1/2 levels at baseline in ONHAs. In addition, our results also suggest that (+)-pentazocine-mediated suppression of oxidative stress-induced ERK1/2 activation occurs through S1R.

## Discussion

Agonists and antagonists of S1R might offer novel treatment options for a broad range of disorders including neurodegenerative diseases and cancer[7, 8, 12, 39]. Stimulation of S1R protects purified neurons, including RGCs, from cell death under conditions of oxidative, metabolic, and excitotoxic stress[7, 14, 21]. However, S1Rs are expressed in glia as well as neurons, within the brain, spinal cord, and optic nerve[16, 40, 41]. Previous work using brain-derived astrocytes indicates that inhibition of S1R leads to decreased astrocyte reactivity[42]. However, the effects of activation or inhibition of S1R on astrocytes derived from ocular tissues are not well characterized. Multiple studies indicate that glia are critical to the health and survival of neurons[43, 44]. Consistent with these findings, astrocytes can modulate the function of associated RGCs, and changes within ONH astrocytes are among the earliest signs of glaucomatous optic neuropathy[45–47]. Therefore, the results of these analyses are critical to evaluation of S1R as a therapeutic target.

Our results show that stimulation of S1R using the specific agonist, (+)-pentazocine, protects ONHAs from death under conditions of oxidative stress. In addition, we found that (+)-pentazocine treatment suppresses ROS generation and modulates ERK activation within ONHAs. Several studies indicate that (+)-pentazocine acts specifically through the S1R[48, 49]. However, some reports describe non-specific (+)-pentazocine-mediated effects[50]. Our results show that (+)-pentazocine-associated pro-survival responses are blocked by knockdown of S1R. Therefore, we conclude that S1R mediates these effects.

Initial transfections of S1R siRNA were performed using the HeLa cell line. Interestingly, knockdown of S1R within these cultures caused significant cell death at baseline, independent of oxidative stress exposure. Comparison of S1R protein levels between transfected HeLa cells and ONHAs showed similar efficiency of S1R knockdown. However, in contrast to HeLa cells, suppression of S1R expression within ONHAs did not cause significant baseline cell death. Several studies have evaluated the pro-apoptotic and growth-inhibitory effects of S1R inhibition within neoplastic cell types[12, 51]. Reported mechanisms that underlie these effects include direct regulation of apoptosis signaling pathways, ER and oxidative cellular stress responses[22, 23, 52]. Our results support the hypothesis that the S1R may be a cancer-specific therapeutic target. However, knock down of S1R in ONHAs was associated with increased levels of ROS and a higher baseline level of ERK phosphorylation. Thus, although survival of ONHAs was not affected upon knock down of S1R, their *in vivo* functions might still be compromised. Thus, the effects of S1R inhibition on these and other cell types must be considered if S1R inhibition is used systemically in cancer treatment.

Recent reports indicate that S1R agonists, including (+)-pentazocine, enhance ERK1/2 phosphorylation within neurons. For example, studies using (+)-pentazocine-treated cultures of primary RGCs have found an S1R-dependent increase in ERK1/2 phosphorylation following 6 hours of oxygen-glucose deprivation[21]. In addition, studies have shown that the S1R agonist, 4-PPBP, enhances ERK1/2 phosphorylation in primary cultures derived from mixed cortical and hippocampal neurons[25].

Our data, showing S1R-mediated suppression of ERK1/2 activation in optic nerve head astrocytes would seemingly conflict with studies reported from neuronal cultures, as discussed above. Our results indicate that stimulation of S1R using (+)-pentazocine suppresses ERK1/2 activation within optic nerve head-derived astrocytes. We have also shown that (+)-pentazocine inhibits ERK1/2 phosphorylation within primary retinal microglia[28]. Taken together, results suggest that agonist-mediated S1R stimulation leads to differential modulation of ERK activity within primary neurons versus glia.

Overall, results from multiple studies indicate that agonists for S1R function to increase ERK1/2 phosphorylation in some circumstances, but act to decrease ERK1/2 phosphorylation in others. This may seem counter-intuitive, but many drugs, including S1R agonists, can show opposite effects under some circumstances [53, 54]. In addition to cell-type differences, the physio-pathological conditions under which the drug is used, as well as drug concentration at the site of action, can determine the signaling cascades that are engaged.

The activities of basic protein kinase signaling pathways, including ERK1/2, do vary depending upon the cellular model analyzed. For example, within the oligodendroglial cell line, CG4, pharmacological blockade of the ERK1/2 pathway prevents H_2_O_2_-induced cell death[55]. In addition, inhibition of ERK1/2 within glia cells blocks production of neuro-toxic nitric oxide and protects midbrain-derived neurons from degeneration[56]. However, within neurons, glutamate-induced, transient activation of ERK1/2 elicits a prosurvival response, while oxidative stress-induced sustained ERK1/2 activation promotes neuronal death[57]. Overall it is likely that the magnitude and duration of ERK1/2 activation within a given cell type, determines the life or death-promoting capacity of this protein kinase[58]. Our results show that sustained inhibition of ERK1/2 phosphorylation increases the viability of oxidative stress-exposed ONHAs. Therefore, inhibition of ERK phosphorylation is likely one mechanism through which S1R mediates pro-survival effects within ONHAs. However, S1R has been shown to interact with a multitude of intracellular targets. Further studies are needed to address which of the many S1R-mediated effects contributes to promotion of ONHA survival upon treatment with the S1R agonist, (+)-pentazocine.

Despite the apparently opposing cell-type specific effects of S1R on ERK1/2 activity, the S1R agonists are pro-survival in multiple primary cell cultures[21, 25, 27, 59] Moreover, S1R stimulation within optic nerve head astrocytes and retinal microglia results in decreased intracellular ROS generation and decreased release of neurotoxic inflammatory cytokines. Therefore, although S1R agonists may variably modulate ERK1/2 activity within glial cells and RGCs, the overall effect of S1R stimulation within these cell types may be to promote neuroprotection. For example, a multitude of studies indicate that neuroinflammation, mediated by microglia and astrocytes, is a key contributing factor to optic nerve degeneration in glaucoma[60, 61]. S1R agonists might act within both astrocytes and microglia to mitigate neurotoxic inflammatory responses while simultaneously augmenting pro-survival pathways within visual system neurons themselves. If this is the case, activation of S1R would offer a powerful, pluripotent, therapeutic option for neuroprotective treatment in glaucoma. Furthermore, antagonists of S1R generally suppress cellular growth and survival[12, 22, 62]. Our results are consistent with these paradigms.

## Supporting information

S1 Data Set Characterization of cultured primary rat optic nerve head astrocytes (ONHA). (A) Additional representative images showing ONHAs fixed and probed with GFAP (red), OSP (red) and Iba-1 (red). The cells were counterstained with DAPI (blue). (B) Representative images showing DAPI positive cells, GFAP positive cells, OSP positive cells and Iba-1 positive cells were counted to analyze the purity of cultured ONHAs. (C) Original western blots showing that cultured ONHAs express GFAP and S1R, but not Iba-1 and OSP.
(TIF)

S2 Data Set The effect of (+)-pentazocine and H_2_O_2_ on ONHA viability. The original GraphPad data showed that A) PTZ did not cause a significant change in percentage viability compared to the untreated control, B) H_2_O_2_ induced ONHA death in a dose dependent manner, and C) Compared to H_2_O_2_–exposure with no PTZ treatment, the H_2_O_2_-exposed, PTZ-treated ONHAs showed significantly increased viability.
(ZIP)

S3 Data Set Effect of PTZ on ROS generation in H_2_O_2_-exposed ONHAs. Additional representative images of ONHAs treated with 100μM H_2_O_2_ for 24 hours in the presence or absence of PTZ (10μM, 1 hour pretreatment followed by co-treatment). Our data showed that intracellular ROS generation increased when ONHAs were exposed to H_2_O_2_. The ROS generation was inhibited by PTZ.
(TIF)

S4 Data Set Inhibition of ERK1/2 phosphorylation in ONHAs. ONHAs were incubated with 100μM H_2_O_2_ at 37°C for 15 minutes, 30 minutes, 1 hour, 3 hours and 24 hours in the presence or absence of PTZ (10μM, 1 hour pretreatment followed by cotreatment). Original western blots of all 3 batches of experiments showed that phosphorylation of ERK was increased at the 15 minute time point following H_2_O_2_ application, and peaked between 30 minutes and 1 hour. Lysates derived from H_2_O_2_-exposed cells treated with PTZ showed decreased ERK phosphorylation at 1 hour, 3 hour and 24 hour time points.
(TIF)

S5 Data Set S1R knockdown in HeLa cells and ONHAs. (A) HeLa cells were transfected with human scrambled siRNA or with human S1R siRNA. Original western blots of all 3 batches of experiments showed that S1R expression levels were decreased after 3 days and 6 days of S1R siRNA transfection in HeLa cells. (B) ONHAs were transfected with rat scrambled siRNA or with rat S1R siRNA. Original western blots of all 3 batches of experiments showed that S1R expression levels were decreased after 3 days and 6 days of S1R siRNA transfection in ONHAs.
(TIF)

S6 Data Set Knockdown of S1R within ONHAs blocks (+)-pentazocine-mediated suppression of ROS generation. Five days following transfection with S1R siRNA, ONHAs were treated with 100μM H_2_O_2_ for 24 hours in the presence or absence of PTZ (10μM, 1 hour pretreatment followed by co-treatment). Additional representative images show the effect of S1R knockdown on ROS generation in ONHAs.
(TIF)

S7 Data Set Knockdown of S1R within ONHAs blocks the (+)-pentazocine-mediated suppression of ERK1/2 phosphorylation. 5 days after S1R siRNA transfection, ONHA were incubated with 100μM H_2_O_2_ at 37°C for 15 minutes, 30 minutes, 1 hour, 3 hours and 24 hours in the presence or absence of PTZ (10μM, 1 hour pretreatment followed by cotreatment). Original western blots of all 3 batches of experiments showed the effect of PTZ on H_2_O_2_–exposed, S1R siRNA-transfected ONHA.
(TIF)

## References

[1] Hayashi T, Su TP. Sigma-1 receptor chaperones at the ER-mitochondrion interface regulate Ca(2+) signaling and cell survival. Cell. 2007;131(3):596–610. Epub 2007/11/06. doi: 10.1016/j.cell.2007.08.036 . 17981125 10.1016/j.cell.2007.08.036

[2] Mavlyutov TA, Duellman T, Kim HT, Epstein ML, Leese C, Davletov BA, et al Sigma-1 receptor expression in the dorsal root ganglion: Reexamination using a highly specific antibody. Neuroscience. 2016;331:148–57. Epub 2016/06/25. doi: 10.1016/j.neuroscience.2016.06.030 27339730 10.1016/j.neuroscience.2016.06.030 PMC5047027

[3] Alonso G, Phan V, Guillemain I, Saunier M, Legrand A, Anoal M, et al Immunocytochemical localization of the sigma(1) receptor in the adult rat central nervous system. Neuroscience. 2000;97(1):155–70. Epub 2000/04/20. . 10771347 10.1016/s0306-4522(00)00014-2

[4] Whitlock BB, Liu Y, Chang S, Saini P, Ha BK, Barrett TW, et al Initial characterization and autoradiographic localization of a novel sigma/opioid binding site in immune tissues. Journal of neuroimmunology. 1996;67(2):83–96. Epub 1996/07/01. . 8765330 10.1016/0165-5728(96)00041-0

[5] Wolfe SA Jr., Culp SG, De Souza EB. Sigma-receptors in endocrine organs: identification, characterization, and autoradiographic localization in rat pituitary, adrenal, testis, and ovary. Endocrinology. 1989;124(3):1160–72. Epub 1989/03/01. doi: 10.1210/endo-124-3-1160 . 2537173 10.1210/endo-124-3-1160

[6] Su TP, Su TC, Nakamura Y, Tsai SY. The Sigma-1 Receptor as a Pluripotent Modulator in Living Systems. Trends in pharmacological sciences. 2016;37(4):262–78. Epub 2016/02/13. doi: 10.1016/j.tips.2016.01.003 26869505 10.1016/j.tips.2016.01.003 PMC4811735

[7] Smith SB, Duplantier J, Dun Y, Mysona B, Roon P, Martin PM, et al In vivo protection against retinal neurodegeneration by sigma receptor 1 ligand (+)-pentazocine. Invest Ophthalmol Vis Sci. 2008;49(9):4154–61. Epub 2008/05/13. doi: 10.1167/iovs.08-1824 . 18469181 10.1167/iovs.08-1824 PMC2562718

[8] Nguyen L, Kaushal N, Robson MJ, Matsumoto RR. Sigma receptors as potential therapeutic targets for neuroprotection. European journal of pharmacology. 2014;743:42–7. Epub 2014/09/28. doi: 10.1016/j.ejphar.2014.09.022 25261035 10.1016/j.ejphar.2014.09.022 PMC4454619

[9] Francardo V, Bez F, Wieloch T, Nissbrandt H, Ruscher K, Cenci MA. Pharmacological stimulation of sigma-1 receptors has neurorestorative effects in experimental parkinsonism. Brain: a journal of neurology. 2014;137(Pt 7):1998–2014. Epub 2014/04/24. doi: 10.1093/brain/awu107 . 24755275 10.1093/brain/awu107

[10] Ajmo CT Jr., Vernon DO, Collier L, Pennypacker KR, Cuevas J. Sigma receptor activation reduces infarct size at 24 hours after permanent middle cerebral artery occlusion in rats. Current neurovascular research. 2006;3(2):89–98. Epub 2006/05/25. . 16719792 10.2174/156720206776875849

[11] Behensky AA, Yasny IE, Shuster AM, Seredenin SB, Petrov AV, Cuevas J. Stimulation of sigma receptors with afobazole blocks activation of microglia and reduces toxicity caused by amyloid-beta25-35. The Journal of pharmacology and experimental therapeutics. 2013;347(2):458–67. Epub 2013/09/06. doi: 10.1124/jpet.113.208348 . 24006337 10.1124/jpet.113.208348

[12] van Waarde A, Rybczynska AA, Ramakrishnan NK, Ishiwata K, Elsinga PH, Dierckx RA. Potential applications for sigma receptor ligands in cancer diagnosis and therapy. Biochimica et biophysica acta. 2015;1848(10 Pt B):2703–14. Epub 2014/09/01. doi: 10.1016/j.bbamem.2014.08.022 . 25173780 10.1016/j.bbamem.2014.08.022

[13] Maurice T, Su TP. The pharmacology of sigma-1 receptors. Pharmacology & therapeutics. 2009;124(2):195–206. Epub 2009/07/22. doi: 10.1016/j.pharmthera.2009.07.001 19619582 10.1016/j.pharmthera.2009.07.001 PMC2785038

[14] Martin PM, Ola MS, Agarwal N, Ganapathy V, Smith SB. The sigma receptor ligand (+)-pentazocine prevents apoptotic retinal ganglion cell death induced in vitro by homocysteine and glutamate. Brain research Molecular brain research. 2004;123(1–2):66–75. Epub 2004/03/30. doi: 10.1016/j.molbrainres.2003.12.019 . 15046867 10.1016/j.molbrainres.2003.12.019 PMC3742374

[15] Mueller BH 2nd, Park Y, Daudt DR 3rd, Ma HY, Akopova I, Stankowska DL, et al Sigma-1 receptor stimulation attenuates calcium influx through activated L-type Voltage Gated Calcium Channels in purified retinal ganglion cells. Experimental eye research. 2013;107:21–31. Epub 2012/11/28. doi: 10.1016/j.exer.2012.11.002 . 23183135 10.1016/j.exer.2012.11.002

[16] Ola MS, Moore P, El-Sherbeny A, Roon P, Agarwal N, Sarthy VP, et al Expression pattern of sigma receptor 1 mRNA and protein in mammalian retina. Brain research Molecular brain research. 2001;95(1–2):86–95. Epub 2001/11/01. . 11687279 10.1016/s0169-328x(01)00249-2 PMC3742362

[17] Ha Y, Saul A, Tawfik A, Williams C, Bollinger K, Smith R, et al Late-onset inner retinal dysfunction in mice lacking sigma receptor 1 (sigmaR1). Investigative ophthalmology & visual science. 2011;52(10):7749–60. Epub 2011/08/25. doi: 10.1167/iovs.11-8169 21862648 10.1167/iovs.11-8169 PMC3183986

[18] Wang J, Saul A, Roon P, Smith SB. Activation of the molecular chaperone, sigma 1 receptor, preserves cone function in a murine model of inherited retinal degeneration. Proc Natl Acad Sci U S A. 2016;113(26):E3764–72. Epub 2016/06/15. doi: 10.1073/pnas.1521749113 27298364 10.1073/pnas.1521749113 PMC4932934

[19] Mavlyutov TA, Nickells RW, Guo LW. Accelerated retinal ganglion cell death in mice deficient in the Sigma-1 receptor. Molecular vision. 2011;17:1034–43. Epub 2011/05/05. 21541278 PMC3084245

[20] Zhao J, Mysona BA, Qureshi A, Kim L, Fields T, Gonsalvez GB, et al (+)-Pentazocine Reduces NMDA-Induced Murine Retinal Ganglion Cell Death Through a sigmaR1-Dependent Mechanism. Invest Ophthalmol Vis Sci. 2016;57(2):453–61. Epub 2016/02/13. doi: 10.1167/iovs.15-18565 26868747 10.1167/iovs.15-18565 PMC4758298

[21] Mueller BH 2nd, Park Y, Ma HY, Dibas A, Ellis DZ, Clark AF, et al Sigma-1 receptor stimulation protects retinal ganglion cells from ischemia-like insult through the activation of extracellular-signal-regulated kinases 1/2. Experimental eye research. 2014;128:156–69. Epub 2014/10/12. doi: 10.1016/j.exer.2014.10.007 . 25305575 10.1016/j.exer.2014.10.007

[22] Das D, Persaud L, Dejoie J, Happy M, Brannigan O, De Jesus D, et al Tumor necrosis factor-related apoptosis-inducing ligand (TRAIL) activates caspases in human prostate cancer cells through sigma 1 receptor. Biochemical and biophysical research communications. 2016;470(2):319–23. Epub 2016/01/23. doi: 10.1016/j.bbrc.2016.01.055 . 26792723 10.1016/j.bbrc.2016.01.055

[23] Happy M, Dejoie J, Zajac CK, Cortez B, Chakraborty K, Aderemi J, et al Sigma 1 Receptor antagonist potentiates the anti-cancer effect of p53 by regulating ER stress, ROS production, Bax levels, and caspase-3 activation. Biochemical and biophysical research communications. 2015;456(2):683–8. Epub 2014/12/17. doi: 10.1016/j.bbrc.2014.12.029 . 25511708 10.1016/j.bbrc.2014.12.029

[24] Wortzel I, Seger R. The ERK Cascade: Distinct Functions within Various Subcellular Organelles. Genes & cancer. 2011;2(3):195–209. Epub 2011/07/23. doi: 10.1177/1947601911407328 21779493 10.1177/1947601911407328 PMC3128630

[25] Tan F G-A P, Downes C, Zhang M, O'Donovan L, Callaway JK, Crack PJ. The σ 1 receptor agonist 4-PPBP elicits ERK1/2 phosphorylation in primary neurons: a possible mechanism of neuroprotective action. Neuropharmacology. 2010;59(6):416–24. doi: 10.1016/j.neuropharm.2010.05.014 20538010 10.1016/j.neuropharm.2010.05.014

[26] Moriguchi S, Yamamoto Y, Ikuno T, Fukunaga K. Sigma-1 receptor stimulation by dehydroepiandrosterone ameliorates cognitive impairment through activation of CaM kinase II, protein kinase C and extracellular signal-regulated kinase in olfactory bulbectomized mice. Journal of neurochemistry. 2011;117(5):879–91. Epub 2011/03/26. doi: 10.1111/j.1471-4159.2011.07256.x . 21434925 10.1111/j.1471-4159.2011.07256.x

[27] Tuerxun T N T, Adachi N, Kumamaru E, Kitazawa H, Kudo M, Kunugi H. SA4503, a sigma-1 receptor agonist, prevents cultured cortical neurons from oxidative stress-induced cell death via suppression of MAPK pathway activation and glutamate receptor expression. Neurosci Lett. 2010;469(3):303–8. doi: 10.1016/j.neulet.2009.12.013 20025928 10.1016/j.neulet.2009.12.013

[28] Zhao J, Ha Y, Liou GI, Gonsalvez GB, Smith SB, Bollinger KE. Sigma receptor ligand, (+)-pentazocine, suppresses inflammatory responses of retinal microglia. Invest Ophthalmol Vis Sci. 2014;55(6):3375–84. Epub 2014/05/09. doi: 10.1167/iovs.13-12823 24812552 10.1167/iovs.13-12823 PMC4042630

[29] Kwon YH, Fingert JH, Kuehn MH, Alward WL. Primary open-angle glaucoma. The New England journal of medicine. 2009;360(11):1113–24. Epub 2009/03/13. doi: 10.1056/NEJMra0804630 19279343 10.1056/NEJMra0804630 PMC3700399

[30] Calkins DJ. Critical pathogenic events underlying progression of neurodegeneration in glaucoma. Progress in retinal and eye research. 2012;31(6):702–19. Epub 2012/08/09. doi: 10.1016/j.preteyeres.2012.07.001 22871543 10.1016/j.preteyeres.2012.07.001 PMC3472111

[31] Hernandez MR. The optic nerve head in glaucoma: role of astrocytes in tissue remodeling. Progress in retinal and eye research. 2000;19(3):297–321. Epub 2000/04/05. . 10749379 10.1016/s1350-9462(99)00017-8

[32] Mandal A, Shahidullah M, Delamere NA. Hydrostatic pressure-induced release of stored calcium in cultured rat optic nerve head astrocytes. Invest Ophthalmol Vis Sci. 51(6):3129–38. Epub 2010/01/15. doi: 10.1167/iovs.09-4614 . 20071675 10.1167/iovs.09-4614 PMC2891472

[33] Beckel JM, Argall AJ, Lim JC, Xia J, Lu W, Coffey EE, et al Mechanosensitive release of adenosine 5'-triphosphate through pannexin channels and mechanosensitive upregulation of pannexin channels in optic nerve head astrocytes: a mechanism for purinergic involvement in chronic strain. Glia. 2014;62(9):1486–501. Epub 2014/05/20. doi: 10.1002/glia.22695 24839011 10.1002/glia.22695 PMC4133947

[34] Jiang G, Mysona B, Dun Y, Gnana-Prakasam JP, Pabla N, Li W, et al Expression, subcellular localization, and regulation of sigma receptor in retinal muller cells. Investigative ophthalmology & visual science. 2006;47(12):5576–82. Epub 2006/11/24. doi: 10.1167/iovs.06-0608 17122151 10.1167/iovs.06-0608 PMC3724475

[35] Wang J, Shanmugam A, Markand S, Zorrilla E, Ganapathy V, Smith SB. Sigma 1 receptor regulates the oxidative stress response in primary retinal Muller glial cells via NRF2 signaling and system xc(-), the Na(+)-independent glutamate-cystine exchanger. Free radical biology & medicine. 2015;86:25–36. Epub 2015/04/30. doi: 10.1016/j.freeradbiomed.2015.04.009 25920363 10.1016/j.freeradbiomed.2015.04.009 PMC4554890

[36] Sofroniew MV. Molecular dissection of reactive astrogliosis and glial scar formation. Trends in neurosciences. 2009;32(12):638–47. Epub 2009/09/29. doi: 10.1016/j.tins.2009.08.002 19782411 10.1016/j.tins.2009.08.002 PMC2787735

[37] Ouyang YB, Voloboueva LA, Xu LJ, Giffard RG. Selective dysfunction of hippocampal CA1 astrocytes contributes to delayed neuronal damage after transient forebrain ischemia. The Journal of neuroscience: the official journal of the Society for Neuroscience. 2007;27(16):4253–60. Epub 2007/04/20. doi: 10.1523/JNEUROSCI.0211-07.2007 17442809 10.1523/JNEUROSCI.0211-07.2007 PMC3140959

[38] Rosenberger J, Petrovics G, Buzas B. Oxidative stress induces proorphanin FQ and proenkephalin gene expression in astrocytes through p38- and ERK-MAP kinases and NF-kappaB. Journal of neurochemistry. 2001;79(1):35–44. Epub 2001/10/12. . 11595755 10.1046/j.1471-4159.2001.00520.x

[39] Francardo V. Sigma-1 receptor: a potential new target for Parkinson's disease? Neural regeneration research. 2014;9(21):1882–3. Epub 2015/01/06. doi: 10.4103/1673-5374.145351 25558236 10.4103/1673-5374.145351 PMC4281425

[40] Moon JY, Roh DH, Yoon SY, Choi SR, Kwon SG, Choi HS, et al sigma1 receptors activate astrocytes via p38 MAPK phosphorylation leading to the development of mechanical allodynia in a mouse model of neuropathic pain. British journal of pharmacology. 2014;171(24):5881–97. Epub 2014/08/28. doi: 10.1111/bph.12893 25158784 10.1111/bph.12893 PMC4290724

[41] Malik M, Rangel-Barajas C, Sumien N, Su C, Singh M, Chen Z, et al The effects of sigma (sigma1) receptor-selective ligands on muscarinic receptor antagonist-induced cognitive deficits in mice. British journal of pharmacology. 2015;172(10):2519–31. Epub 2015/01/13. doi: 10.1111/bph.13076 25573298 10.1111/bph.13076 PMC4409904

[42] Zhang Y, Lv X, Bai Y, Zhu X, Wu X, Chao J, et al Involvement of sigma-1 receptor in astrocyte activation induced by methamphetamine via up-regulation of its own expression. Journal of neuroinflammation. 2015;12:29 Epub 2015/04/19. doi: 10.1186/s12974-015-0250-7 25889537 10.1186/s12974-015-0250-7 PMC4340104

[43] Markiewicz I, Lukomska B. The role of astrocytes in the physiology and pathology of the central nervous system. Acta neurobiologiae experimentalis. 2006;66(4):343–58. Epub 2007/02/03. . 17265695 10.55782/ane-2006-1623

[44] Kimelberg HK, Nedergaard M. Functions of astrocytes and their potential as therapeutic targets. Neurotherapeutics: the journal of the American Society for Experimental NeuroTherapeutics. 2010;7(4):338–53. Epub 2010/10/01. doi: 10.1016/j.nurt.2010.07.006 20880499 10.1016/j.nurt.2010.07.006 PMC2982258

[45] Newman EA, Zahs KR. Modulation of neuronal activity by glial cells in the retina. The Journal of neuroscience: the official journal of the Society for Neuroscience. 1998;18(11):4022–8. Epub 1998/06/06. 9592083 10.1523/JNEUROSCI.18-11-04022.1998 PMC2904245

[46] Schneider M, Fuchshofer R. The role of astrocytes in optic nerve head fibrosis in glaucoma. Experimental eye research. 2016;142:49–55. Epub 2015/09/01. doi: 10.1016/j.exer.2015.08.014 . 26321510 10.1016/j.exer.2015.08.014

[47] Tehrani S, Johnson EC, Cepurna WO, Morrison JC. Astrocyte processes label for filamentous actin and reorient early within the optic nerve head in a rat glaucoma model. Invest Ophthalmol Vis Sci. 2014;55(10):6945–52. Epub 2014/09/27. doi: 10.1167/iovs.14-14969 25257054 10.1167/iovs.14-14969 PMC4215744

[48] de Costa BR, Bowen WD, Hellewell SB, Walker JM, Thurkauf A, Jacobson AE, et al Synthesis and evaluation of optically pure [3H]-(+)-pentazocine, a highly potent and selective radioligand for sigma receptors. FEBS letters. 1989;251(1–2):53–8. Epub 1989/07/17. . 2568952 10.1016/0014-5793(89)81427-9

[49] Langa F, Codony X, Tovar V, Lavado A, Gimenez E, Cozar P, et al Generation and phenotypic analysis of sigma receptor type I (sigma 1) knockout mice. The European journal of neuroscience. 2003;18(8):2188–96. Epub 2003/11/19. . 14622179 10.1046/j.1460-9568.2003.02950.x

[50] Kume T, Nishikawa H, Taguchi R, Hashino A, Katsuki H, Kaneko S, et al Antagonism of NMDA receptors by sigma receptor ligands attenuates chemical ischemia-induced neuronal death in vitro. European journal of pharmacology. 2002;455(2–3):91–100. Epub 2002/11/26. . 12445574 10.1016/s0014-2999(02)02582-7

[51] Kim FJ, Schrock JM, Spino CM, Marino JC, Pasternak GW. Inhibition of tumor cell growth by Sigma1 ligand mediated translational repression. Biochemical and biophysical research communications. 2012;426(2):177–82. Epub 2012/08/29. doi: 10.1016/j.bbrc.2012.08.052 22925888 10.1016/j.bbrc.2012.08.052 PMC3480203

[52] Do W, Herrera C, Mighty J, Shumskaya M, Redenti SM, Sauane M. Sigma 1 Receptor plays a prominent role in IL-24-induced cancer-specific apoptosis. Biochemical and biophysical research communications. 2013;439(2):215–20. Epub 2013/08/31. doi: 10.1016/j.bbrc.2013.08.057 . 23988449 10.1016/j.bbrc.2013.08.057

[53] Cobos EJ, Entrena JM, Nieto FR, Cendan CM, Del Pozo E. Pharmacology and therapeutic potential of sigma(1) receptor ligands. Current neuropharmacology. 2008;6(4):344–66. Epub 2009/07/10. doi: 10.2174/157015908787386113 19587856 10.2174/157015908787386113 PMC2701284

[54] Hayashi T, Maurice T, Su TP. Ca(2+) signaling via sigma(1)-receptors: novel regulatory mechanism affecting intracellular Ca(2+) concentration. The Journal of pharmacology and experimental therapeutics. 2000;293(3):788–98. Epub 2000/06/28. . 10869377

[55] Bhat NR, Zhang P. Hydrogen peroxide activation of multiple mitogen-activated protein kinases in an oligodendrocyte cell line: role of extracellular signal-regulated kinase in hydrogen peroxide-induced cell death. Journal of neurochemistry. 1999;72(1):112–9. Epub 1999/01/14. . 9886061 10.1046/j.1471-4159.1999.0720112.x

[56] Canals S, Casarejos MJ, de Bernardo S, Solano RM, Mena MA. Selective and persistent activation of extracellular signal-regulated protein kinase by nitric oxide in glial cells induces neuronal degeneration in glutathione-depleted midbrain cultures. Molecular and cellular neurosciences. 2003;24(4):1012–26. Epub 2003/12/31. . 14697665 10.1016/j.mcn.2003.08.004

[57] Luo Y, DeFranco DB. Opposing roles for ERK1/2 in neuronal oxidative toxicity: distinct mechanisms of ERK1/2 action at early versus late phases of oxidative stress. J Biol Chem. 2006;281(24):16436–42. Epub 2006/04/20. doi: 10.1074/jbc.M512430200 . 16621802 10.1074/jbc.M512430200

[58] Subramaniam S, Unsicker K. ERK and cell death: ERK1/2 in neuronal death. The FEBS journal. 2010;277(1):22–9. Epub 2009/10/22. doi: 10.1111/j.1742-4658.2009.07367.x . 19843173 10.1111/j.1742-4658.2009.07367.x

[59] Dun Y, Thangaraju M, Prasad P, Ganapathy V, Smith SB. Prevention of excitotoxicity in primary retinal ganglion cells by (+)-pentazocine, a sigma receptor-1 specific ligand. Investigative ophthalmology & visual science. 2007;48(10):4785–94. Epub 2007/09/28. doi: 10.1167/iovs.07-0343 17898305 10.1167/iovs.07-0343 PMC3742388

[60] Williams PA, Marsh-Armstrong N, Howell GR. Neuroinflammation in glaucoma: A new opportunity. Experimental eye research. 2017 Epub 2017/03/01. doi: 10.1016/j.exer.2017.02.014 . 28242160 10.1016/j.exer.2017.02.014 PMC5497582

[61] Vargas JL, Di Polo A. Neuroinflammation in glaucoma: soluble tumor necrosis factor alpha and the connection with excitotoxic damage. Neural regeneration research. 2016;11(3):424–6. Epub 2016/04/30. doi: 10.4103/1673-5374.179053 27127480 10.4103/1673-5374.179053 PMC4829006

[62] Wang L, Prescott AR, Spruce BA, Sanderson J, Duncan G. Sigma receptor antagonists inhibit human lens cell growth and induce pigmentation. Invest Ophthalmol Vis Sci. 2005;46(4):1403–8. Epub 2005/03/26. doi: 10.1167/iovs.04-1209 . 15790908 10.1167/iovs.04-1209

