# Supplementary figures and images for: Sigma 1 receptor regulates ERK activation and promotes survival of optic nerve head astrocytes
Source: PLoS One. 2017 Sep 12;12(9):e0184421. doi: 10.1371/journal.pone.0184421 (PMC5595338; doi:10.1371/journal.pone.0184421)

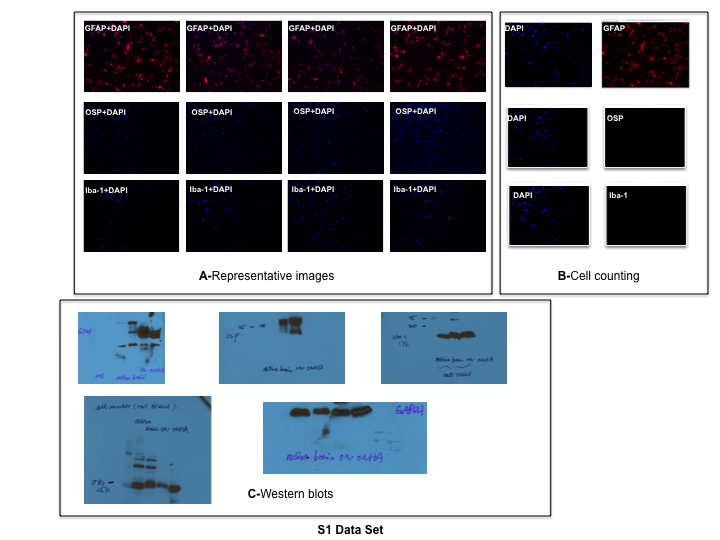

Supplement: S1 Data Set — (A) Additional representative images showing ONHAs fixed and probed with GFAP (red), OSP (red) and Iba-1 (red). The cells were counterstained with DAPI (blue). (B) Representative images showing DAPI positive cells, GFAP positive cells, OSP positive cells and Iba-1 positive cells were counted to analyze the purity of cultured ONHAs. (C) Original western blots showing that cultured ONHAs express GFAP and S1R, but not Iba-1 and OSP. (TIF) [file pone.0184421.s001.tif]

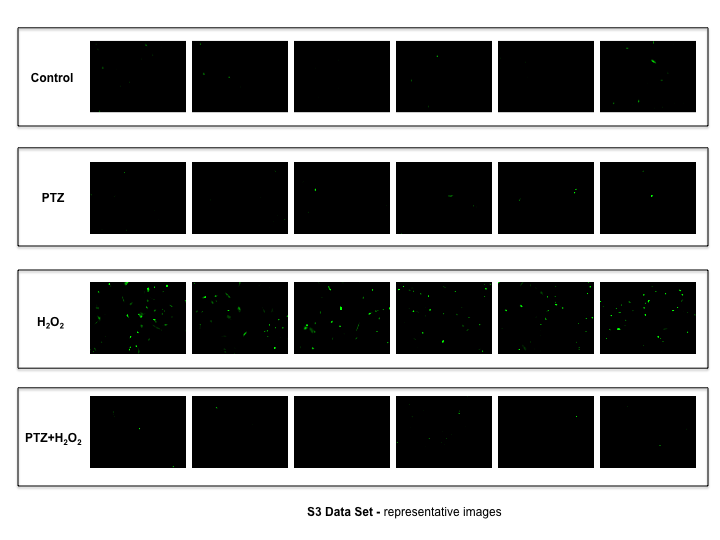

Supplement: S3 Data Set — Additional representative images of ONHAs treated with 100μM H2O2 for 24 hours in the presence or absence of PTZ (10μM, 1 hour pretreatment followed by co-treatment). Our data showed that intracellular ROS generation increased when ONHAs were exposed to H2O2. The ROS generation was inhibited by PTZ. (TIF) [file pone.0184421.s003.tif]

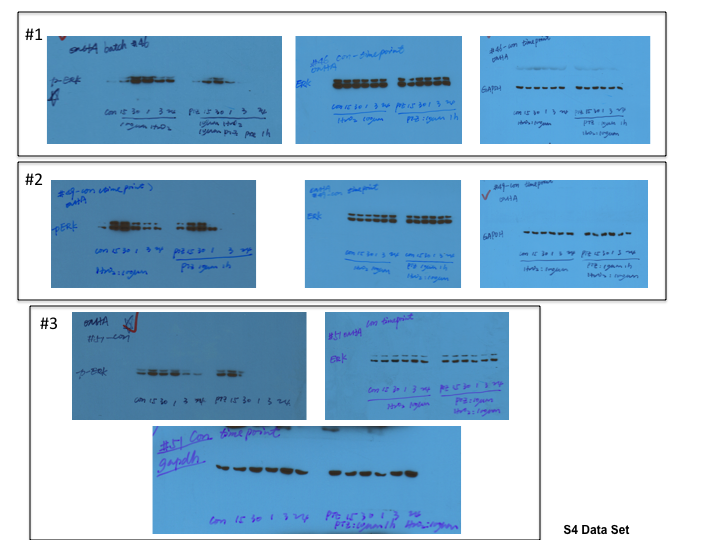

Supplement: S4 Data Set — ONHAs were incubated with 100μM H2O2 at 37°C for 15 minutes, 30 minutes, 1 hour, 3 hours and 24 hours in the presence or absence of PTZ (10μM, 1 hour pretreatment followed by cotreatment). Original western blots of all 3 batches of experiments showed that phosphorylation of ERK was increased at the 15 minute time point following H2O2 application, and peaked between 30 minutes and 1 hour. Lysates derived from H2O2-exposed cells treated with PTZ showed decreased ERK phosphorylation at 1 hour, 3 hour and 24 hour time points. (TIF) [file pone.0184421.s004.tif]

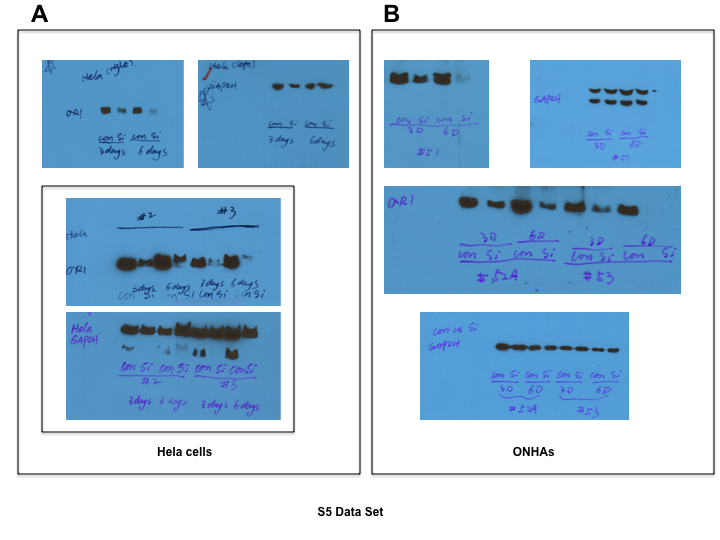

Supplement: S5 Data Set — (A) HeLa cells were transfected with human scrambled siRNA or with human S1R siRNA. Original western blots of all 3 batches of experiments showed that S1R expression levels were decreased after 3 days and 6 days of S1R siRNA transfection in HeLa cells. (B) ONHAs were transfected with rat scrambled siRNA or with rat S1R siRNA. Original western blots of all 3 batches of experiments showed that S1R expression levels were decreased after 3 days and 6 days of S1R siRNA transfection in ONHAs. (TIF) [file pone.0184421.s005.tif]

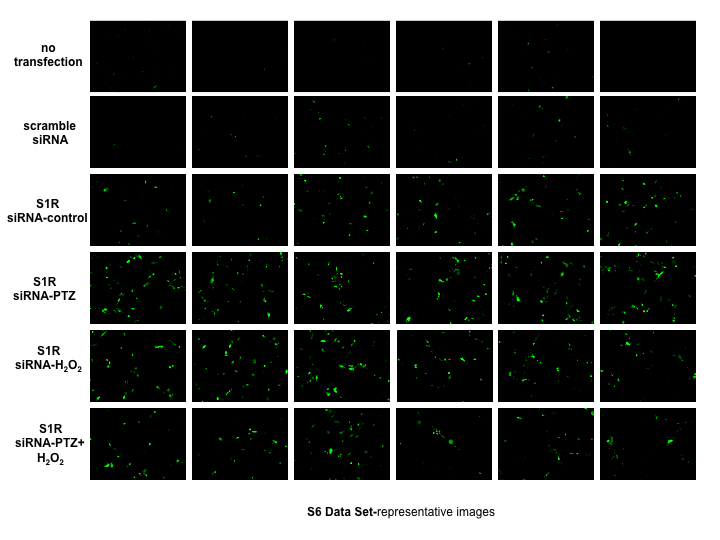

Supplement: S6 Data Set — Five days following transfection with S1R siRNA, ONHAs were treated with 100μM H2O2 for 24 hours in the presence or absence of PTZ (10μM, 1 hour pretreatment followed by co-treatment). Additional representative images show the effect of S1R knockdown on ROS generation in ONHAs. (TIF) [file pone.0184421.s006.tif]

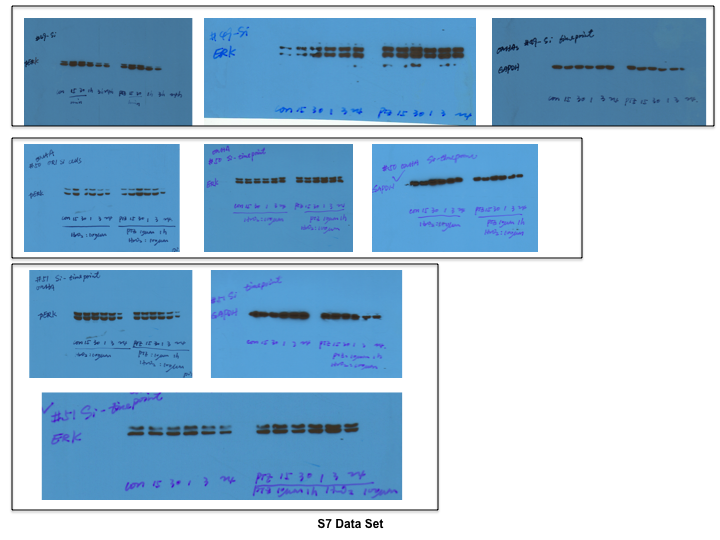

Supplement: S7 Data Set — 5 days after S1R siRNA transfection, ONHA were incubated with 100μM H2O2 at 37°C for 15 minutes, 30 minutes, 1 hour, 3 hours and 24 hours in the presence or absence of PTZ (10μM, 1 hour pretreatment followed by cotreatment). Original western blots of all 3 batches of experiments showed the effect of PTZ on H2O2–exposed, S1R siRNA-transfected ONHA. (TIF) [file pone.0184421.s007.tif]
